# Green Synthesis of Boron Carbonitride with High Capacitance

**DOI:** 10.3390/ma11030387

**Published:** 2018-03-06

**Authors:** Dongping Chen, Yanzhen Huang, Xinling Hu, Rongkai Li, Yingjiang Qian, Dongxu Li

**Affiliations:** College of Materials Science and Engineering, Huaqiao University, Xiamen 361021, China; 1511302040@hqu.edu.cn (D.C.); 15737567342@163.com (Y.H.); 18224566342@163.com (X.H.); liyungkai@yeah.net (R.L.); qian1358_love@163.com (Y.Q.)

**Keywords:** solvothermal, boron carbonitride, supercapacitors, Microstructure

## Abstract

Boron carbonitrides (BCN) have attracted great interest in superhard or energy storage materials. In this work, thin BCN sheets were synthesized at 250 °C by a facile and green solvothermal method. The structure and morphology were characterized by X-ray diffraction (XRD), Raman spectroscopy, X-ray photoelectron spectroscopy (XPS), scanning electron microscopy (SEM), and transmission electron microscopy (TEM). Based on the results of electrochemical experiments, the thin BCN sheet exhibited excellent capacitance performance (343.1 F/g at a current density of 0.5 A/g) and cycling stability (90%), which showed high potential applications in supercapacitors.

## 1. Introduction

Capacitance (C) and Boron Nitride (BN) have similar atomic structures and physical properties but different thermal stability and electrical conductivity [1,2]. Ternary boron carbonitrides (BCN) compounds were produced using C and BN, which exhibit favorable properties particularly excellent force, heat, electricity and light [3,4,5]. These materials are considered as products of the substitution of some carbon atoms in the graphite network with boron (B) or Nitrogen (N) atoms [6]. Ternary BCN nanotubes possess outstanding mechanical and electrical properties as compared with carbon nanotubes, good thermal and chemical stability, even under extreme conditions, such as high temperature and high pressure, although their morphologies are similar [7,8]. Furthermore, the diameter of a ternary BCN compound has minimal effect on its mechanical and electrical properties. Therefore, BCN materials in composite, magnetic, luminescent, and electronic materials have a wide range of applications [9,10].

Although BCN compounds have many excellent properties, it is difficult to synthesize and synthesis yields is low. At present, BCN materials were prepared by the arc discharge, ion beam sputtering, chemical vapor deposition, and high temperature and high pressure (HTHP) methods [11,12]. Zeng et al. [13] prepared boron carbonitride microspheres by an organic precursor pyrolysis approach. Qin et al. [14] reported an approach to synthesize few atomic layered BCN sheets by CVD method. Among them, HTHP method is the most widely used one to synthesize metastable BCN. Therefore, it is necessary to find a simple and low-cost process for the development of BCN materials with excellent physical and chemical properties through mild reaction conditions.

In this study, thin BCN sheets (1–50 μm) were synthesized through the solvothermal method. The results of electrochemical experiments showed that the synthesized BCN materials exhibit ultrahigh specific capacitance and good cycle stability, which are valuable in terms of supercapacitor applications.

## 2. Materials and Methods

### 2.1. Synthesis of Thin BCN Sheets 

Boric acid (1.76 g) and 2,4,6-Tri(2-pyridyl)-1,3,5-triazine (0.5 g) was dissolved in ethylene glycol and ultrasonic treatment for 10 min. The yellow solution that was obtained was transferred to a hydrothermal reactor. The reactor was transferred to a stove and heated up to 250 °C for 24 h. After cooling, the black and viscous liquid was collected and placed in a corundum boat, which was then placed in tube furnace and heated at a rate of 10 °C/min to 400 °C for 2 h under N_2_ atmosphere for removing the organic solvent. The boat was then allowed to cool at room temperature. The products were collected and washed with hydrochloric acid (5%) and distilled water to remove the boron salt and other impurities. Then, the final product was dried in a vacuum at 80 °C for 8 h.

### 2.2. Characterization

X-ray powder diffraction (XRD) patterns were collected on a Rigaku MiniFlex 600 with Cu-Kα radiation. Raman spectra were collected on a RENISHAW inVia (Freeboard International Co., Ltd., Hong Kong, China) at 532 nm wavelength. Scanning electron microscopy (SEM) images were collected through a Hitachi field-emission scanning electron microscope. The transmission electron microscopy (TEM) images were collected through JEM-2100 (Ruisheng Technology Co., Ltd., Shenzhen, China). at an acceleration voltage of 200 KV. The X-ray photoelectron spectroscopy (XPS) was collected on a Thermo ESCALAB 250 (Thermo Fisher Scientific, Shanghai, China), with an X-ray Al Kα source. After correction, the binding energy of the main peak C 1s in the sample was found to be certain at 284.8 eV.

## 3. Results and Discussion

**Figure 1:**
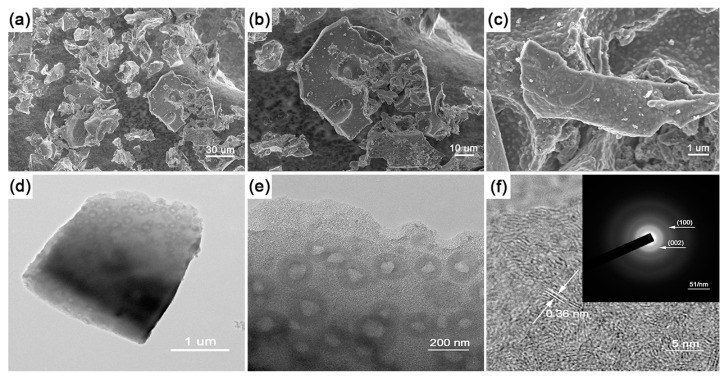
Scanning electron microscopy (SEM) images of thin boron carbonitrides (BCN) sheets (**a**–**c**); transmission electron microscopy (TEM) image (**d**–**e**); and, high-resolution transmission electron microscopy (HRTEM) (**f**) image with selected area electron diffraction (SEAD) pattern (inset) of thin BCN sheets.

The SEM and TEM images of the BCN samples were obtained to characterize their morphology and structures. Figure 1a–c shows the SEM image of the product. The samples were observed with irregularly flaky shape existing small blisters on the surface (Figure 1c,e), which might be formed from the vaporization of organic solution. Based on typical high-resolution transmission electron microscopy (HRTEM) and selected area electron diffraction (SAED), the lattice spacing (d) calculated is about 0.36 nm, which corresponded to the XRD diffraction pattern (002). The EDS and Elemental Mapping of BCN samples were obtained to explain the distribution of elements. (In Supplementary Materials, Figure S2).

**Figure 2:**
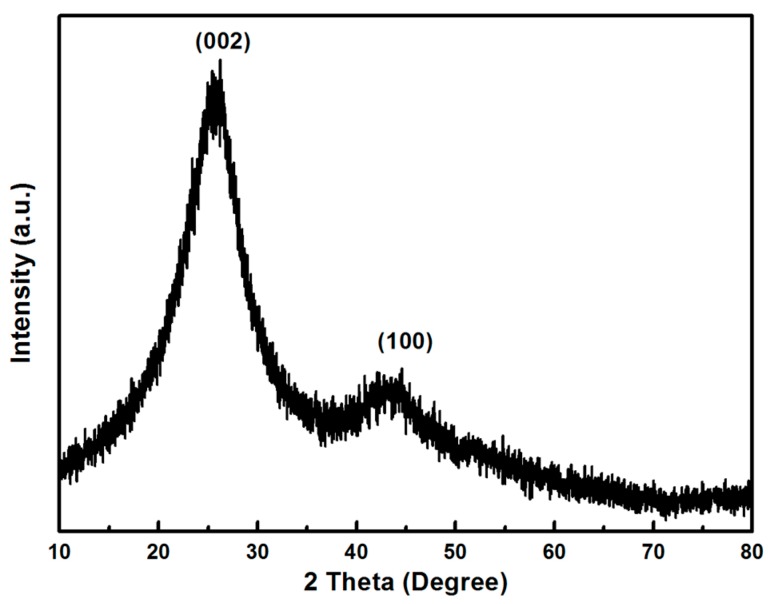
X-ray diffraction (XRD) patterns of thin BCN sheets.

Thin BCN sheet materials were synthesized by a facile solvothermal method of boric acid, 2,4,6-Tri(2-pyridyl)-1,3,5-triazine, and ethylene glycol in the reactor. Figure 2 shows the XRD pattern of the BCN samples. Two peaks were observed for the BCN samples, particularly the peaks of ~26° and ~43°, which are related to the (002) and (100) planes of the graphite-like structure of BCN, respectively [15,16].

**Figure 3:**
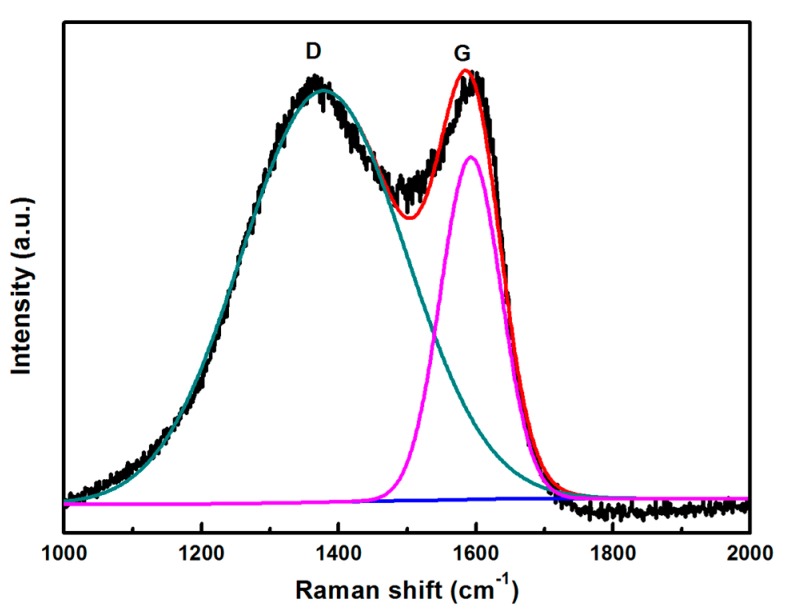
Raman spectra of thin BCN sheets.

The Raman spectra of the BCN samples (Figure 3) were examined to understand their physical and chemical properties. Two obvious characteristic peaks were observed, namely, the D- and G-bands of graphitic structure at 1377.8 and 1592.6 cm^−1^, respectively. The G-band was generated by the in-plane stretching vibration of all the *sp*^2−^ bonded chemical bonds C-C, C-N, B-N, and B-C [17]. The D-band was generated by *sp*^3^ defects or lattice imperfections [18].

**Figure 4:**
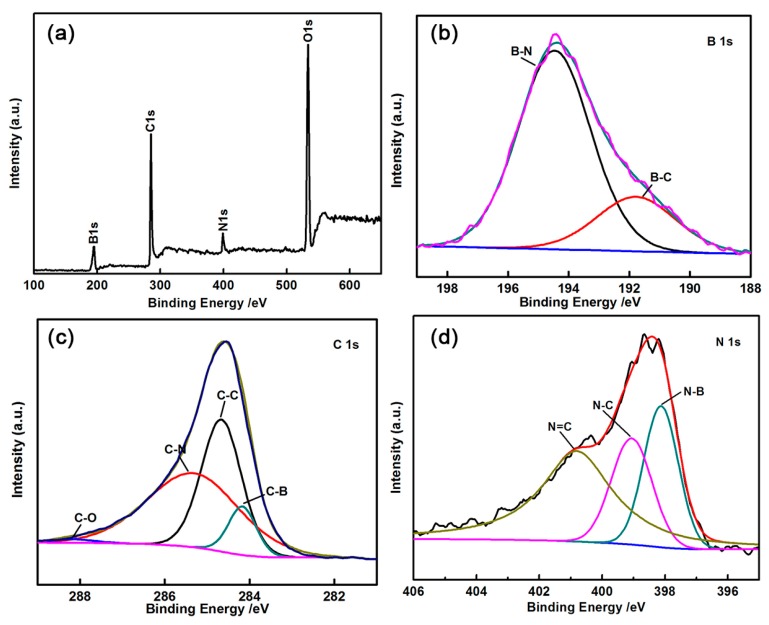
X-ray photoelectron spectroscopy (XPS) scan survey spectra of thin BCN sheets (**a**); B 1s (**b**); C 1s (**c**) and N 1s (**d**).

The XPS spectra and FTIR (In Supplementary Materials, Figure S1) of BCN samples were obtained to investigate their elemental compositions (Figure 4). Figure 4a shows the full range XPS spectra, which were defined as the presence of B, C, N, and O in the BCN samples. The spectrum of B 1s (Figure 4b) can be assigned to two main subpeaks at 191.7 and 194.4 eV, which correspond to B-C and B-N bonds, respectively [19]. As shown in Figure 4c, the high-resolution spectrum of C 1s consists of four subpeaks at 284.1, 284.6, 285.3, and 288.1 eV, which correspond to C-B, C-C, C-N, and C-O bands, respectively [20,21]. As shown in Figure 4d, the signal of the N 1s peaks can be divided into three subpeaks, 398.3, 399.9, and 400.8 eV, which correspond to the N-B bond, N-C bond of the graphite-like structure, and N-C bond of pyridine, respectively [22].

**Figure 5:**
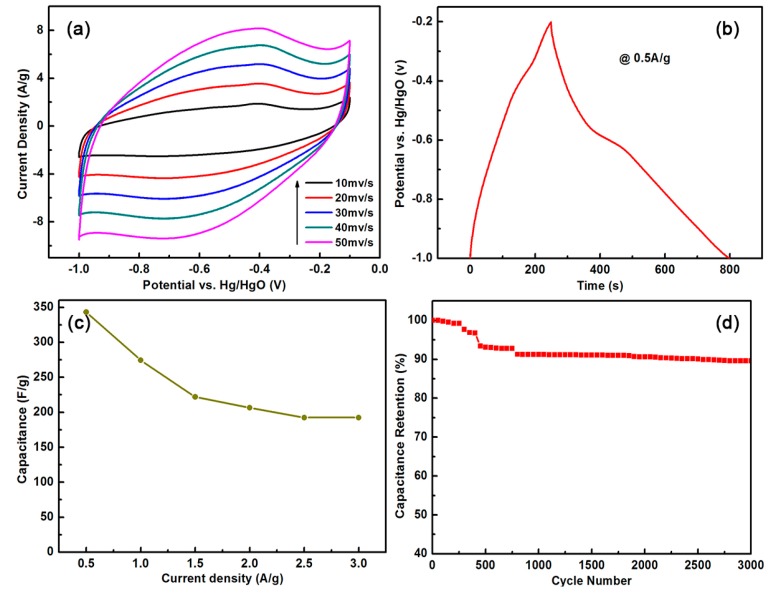
Cyclic voltammetry (CV) curves of thin BCN sheets in 6 M KOH solution at scan rates of 10, 20, 30, 40, and 50 mV/s (**a**); Galvanostatic charge/discharge curves at a current density of 0.5 A/g (**b**); corresponding capacity retention at the current density from 0.5 to 3 A/g (**c**); and, Stability evolution of BCN samples at a current density of 8 A/g in 6 M KOH solution (**d**).

The cyclic voltammetry (CV) and galvanostatic charge/discharge (Figure 5a,b) were used to investigate electrochemical behaviors of thin BCN sheets as electrodes. Figure 5a shows the typical CV curves of thin BCN sheets that were prepared in a 6 M KOH electrolyte for a three electrode cell. The scan rates were 10, 20, 30, 40, and 50 mV/s between −1.0 and −0.1 V. As expected, the distortion of CV curves are mainly due to the sample contains some impurities such as free carbon and carbonitride. Overall, the approximately rectangular CV curve can be observed in the electrochemical double-layer capacitors.

The galvanostatic charge/discharge curve shows that thin BCN sheets have excellent capacitance even at a high-current load (3 A/g). Previous studies showed that doping heteroatom can alter the electronic properties of a carbon material because the heteroatoms tend to attract ions from the electrolyte [23]. Thus, the wettability between the electrode material and electrolyte increases, thus directly increasing the capacitance of the electric double layer of the BCN material. The presence of impurities explained the charge/discharge platform in the Figure 5b. The specific capacitance (*C*) was calculated according to the galvanostatic charge/discharge curve. The formula used was *I∙*∆*t/m∙*∆*v*, where *I*, ∆*t*, *m*, and ∆*v* are the charge/discharge current, discharge time, active substance quality, and the voltage difference, respectively. Figure 5b shows the specific capacitance of the thin BCN sheets prepared according to the galvanostatic charge/discharge curve. In a 6 M KOH solution, the specific capacitance of the BCN sheets was 343.1 F/g when the current density was 0.5 A/g, higher than the those of vertically aligned BCN nanotubes by chemical vapor deposition method (321 F/g at 0.2 A/g) and BCN graphene by thermal annealing method (130.7 F/g at 0.2 A/g), under the same test conditions [24,25]. The high specific capacitance of the BCN sheets may be attributed to the role of heteroatoms (B and N), which form polar covalent bonds that promote ion exchanges between the electrode material and electrolyte. Electrochemical impedance spectroscopy (EIS) was studied for the BCN samples. (In Supplementary Materials, Figure S4)

The rate capability of the BCN sheets was investigated to determine the relationships between capacitance and charge and discharge current density (Figure 5c). The percentage of the specific capacitance and starting specific capacitance (0.5 A/g) at different current densities is defined as the capacitance retention. When the current density increased from 0.5 A/g to 3 A/g, the capacitance retention of the prepared BCN sheets remained at 56%, indicating that the BCN sheets have improved rate performance. Meanwhile, the life cycle test is an important index for the performance evaluation of electrochemical capacitors. Charge and discharge tests were performed on the prepared electrode materials at a current density of 8 A/g for 3000 times. As shown in the Figure 5d, after 3000 times of charge and discharge tests, the electrode can retain about 90% of the efficiency, indicating that the prepared electrode has good electrochemical stability.

## 4. Conclusions

In summary, thin BCN sheets (1–50 µm) were synthesized via a green and facile solvothermal method. Typical peaks of the graphite-like structure of BCN were observed at 26° and 43°. B-C, B-N, C-C and C-N bonds were characterized by XPS spectra, indicating real ternary BCN compounds. Here, heteroatom (B and N) influenced the electronic properties of carbon materials, which increased the wettability of electrode, thereby increasing the specific capacitance of the material. The results of the electrochemical tests showed that the synthesized thin BCN sheets demonstrate extremely high specific capacitance (343.1 F/g when the current density was 0.5 A/g) and good cycle stability (retaining about 90% of the efficiency after 3000 cycles). Thus, thin BCN sheets have high potential applications for supercapacitor.

## Supplementary Materials

The following are available online at http://www.mdpi.com/1996-1944/11/3/387/s1, Figure S1: FTIR of BCN sheets; Figure S2: EDS and Elemental Mapping of BCN sheets; Figure S3: Adsorption and desorption curve of BCN sheets; Figure S4: Nyquist plots of BCN sheets.
