# Supplementary material for: Green Synthesis of Boron Carbonitride with High Capacitance
Source: Materials (Basel). 2018 Mar 6;11(3):387. doi: 10.3390/ma11030387 (PMC5872966; doi:10.3390/ma11030387)

Green Synthesis of Boron Carbonitride with High Capacitance

Dongping Chen, Yanzhen Huang, Xinling Hu, Rongkai Li, Yingjiang Qian, Dongxu Li*

College of Materials Science and Engineering, Huaqiao University, Xiamen, 361021, China

Corresponding Author E-mail: lidongxu@hqu.edu.cn

Contents

Figure S1	FTIR of BCN sheets	S2
Figure S2	EDS and Elemental Mapping of BCN sheets	S2
Figure S3	Adsorption and desorption curve of BCN sheets	S3
Figure S4	Nyquist plots of BCN sheets	S4

Figure S1. FTIR of BCN sheets

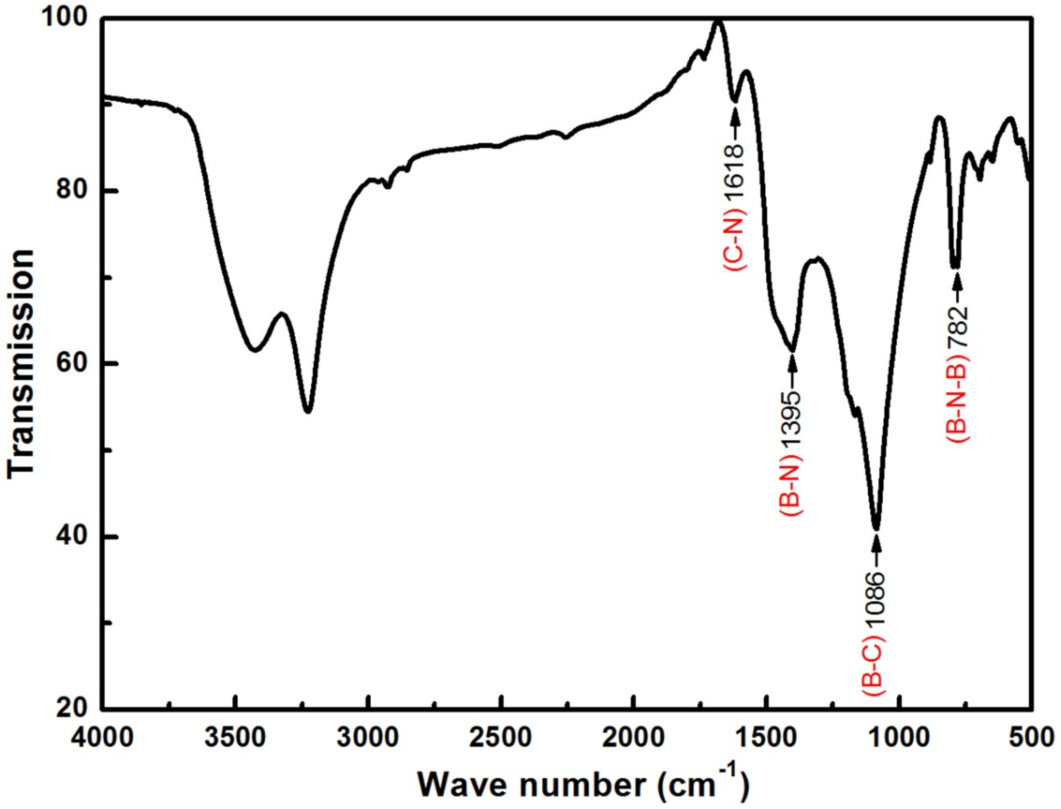

Figure S2. EDS and Elemental Mapping of BCN sheets

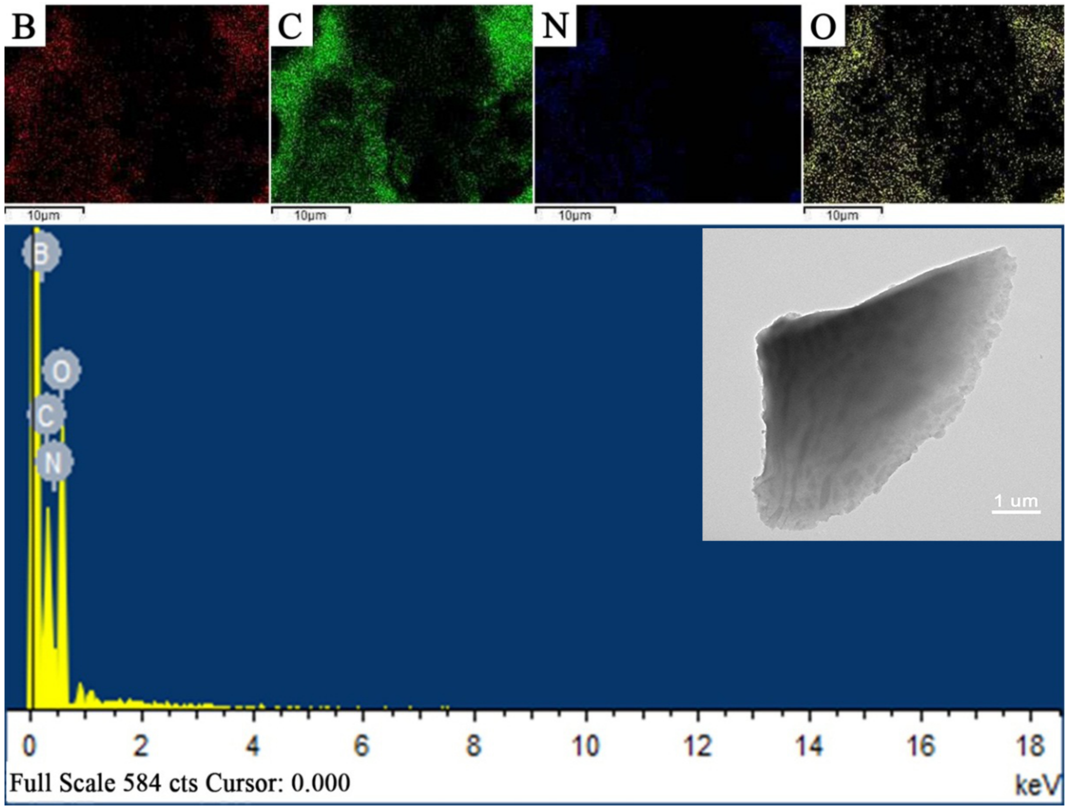

Figure S3. Adsorption and desorption curve of BCN sheets (BET surface area: 158 m^2/g)

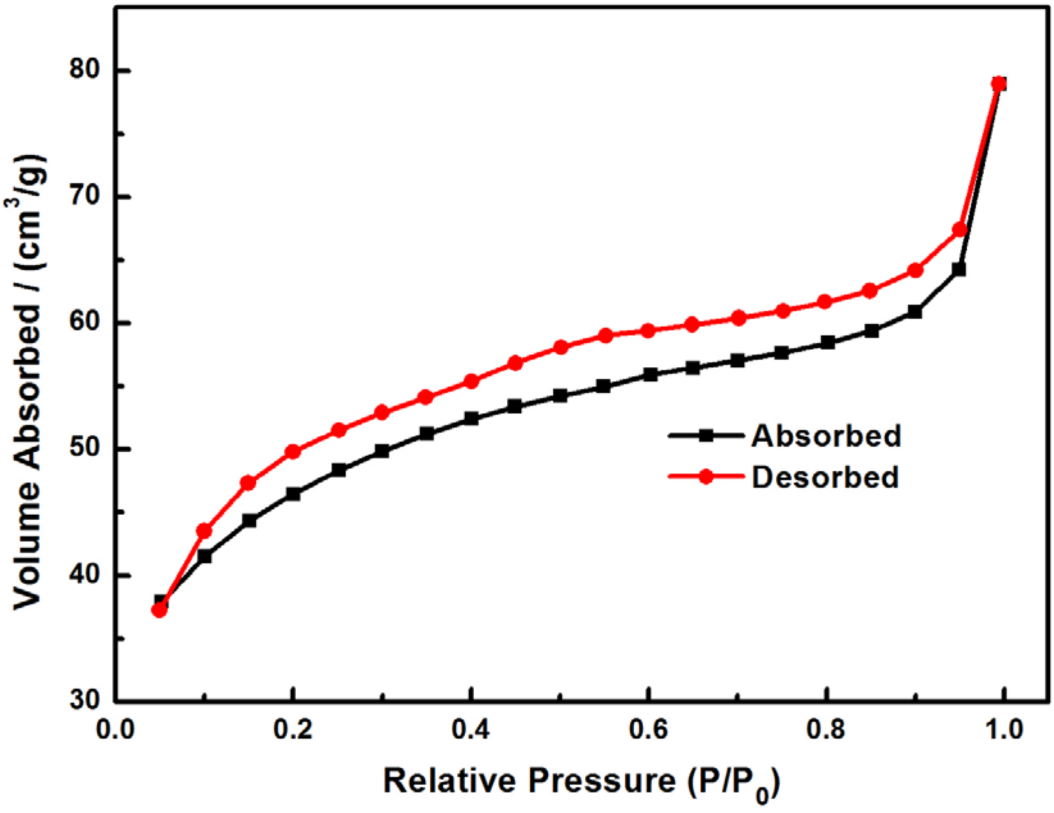

Figure S4. Nyquist plots of BCN sheets

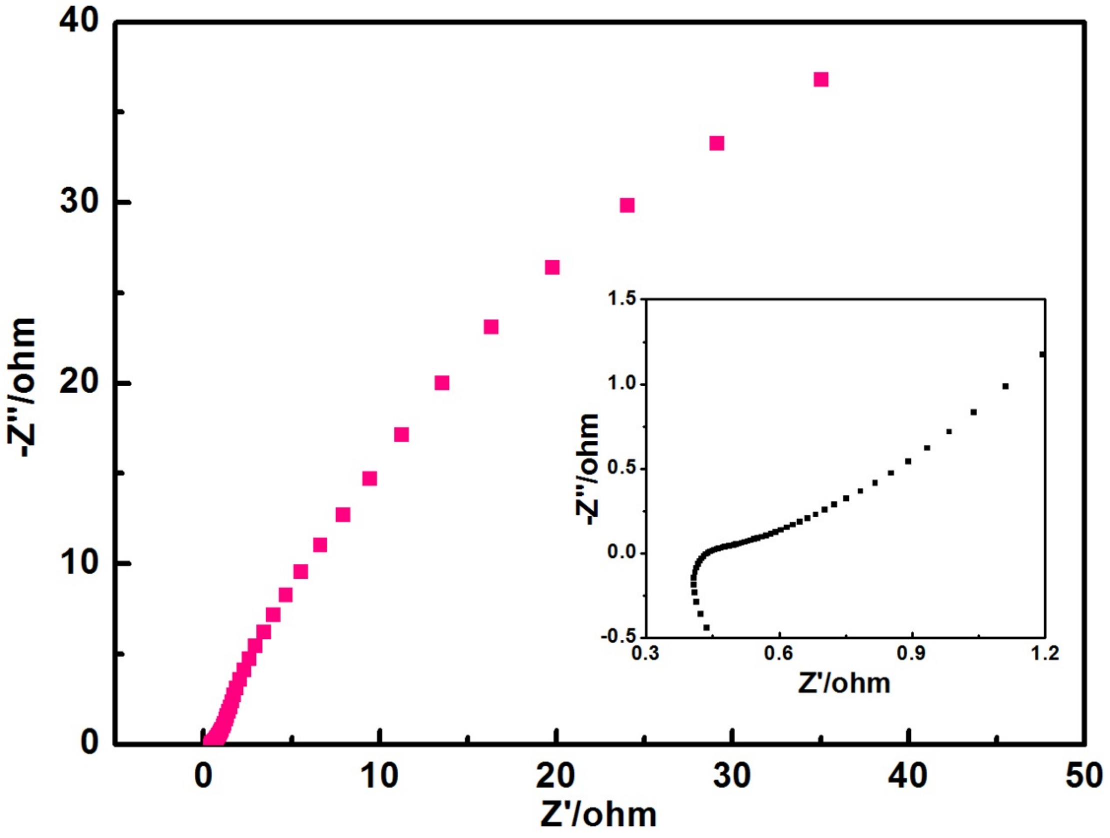

Supplement: Supplementary file 1 [file materials-11-00387-s001.pdf]
